# Being Nice Is Not Enough-Exploring Relationship-Centered Veterinary Care With Structural Equation Modeling. A Quantitative Study on German Pet Owners' Perception

**DOI:** 10.3389/fvets.2019.00056

**Published:** 2019-02-28

**Authors:** Alina M. Küper, Roswitha Merle

**Affiliations:** Department of Veterinary Medicine, Institute for Veterinary Epidemiology and Biostatistics, Freie Universität Berlin, Berlin, Germany

**Keywords:** veterinary medicine, partnership building, relationship-centered care, veterinary-pet owner-communication, empathy, shared decision making, structural equation modeling

## Abstract

During the last years, the philosophy of relationship-centered care gained increasing attention in veterinary medicine. Relationship-centered care is based on a joint venture between pet owner and veterinarians and therefore offers the opportunity to satisfy the pet owners' need for participation in medical decision-making and to provide the best care for the patient. Although research on relationship-centered care in the veterinary consultation is still limited, the available findings suggest that the characteristics of relationship-centered care reflect the pet owners' expectations on satisfactory veterinary care. In this study a quantitative survey was conducted among German pet owners that collected information regarding their perception of the veterinarians' communication during the last appointments. Questionnaires were available online and paper-based. Data were analyzed using exploratory factor analysis and structural equation modeling using SAS. First aim of the study was to explore structural equation modeling (SEM) as an opportunity to evaluate quantitative data in the field of research on relationship-centered care. Further, SEM was used to evaluate associations between the implementation of different characteristics of relationship-centered care in the veterinary practice (empathic communication, partnership-building) and latent outcome variables regarding the pet owners (pet owners' need for further information, consultation of competing health care providers). *N* = 1,270 valid questionnaires were completed. Participants owned small companion animals (55.6%), horses (7.6%), or both (36.9%) within the last 2 years. Results of SEM suggest that partnership-centered and empathic communication decreased the pet owners' needs for further information (e.g., from online sources) and their need to consult alternative health providers (e.g., homeopaths). Especially addressing the pet owners' worries and fears, discussing pros and cons of diagnostic and therapeutic options as well as providing the required amount of information were shown to be large influence factors within the model. Therefore, veterinarians are recommended to implement the concept of partnership-centered care in their daily practice, for it may increase pet owners' loyalty and satisfaction. Results may motivate future research in this field. Further development of the proposed model assumptions may enable valuable progress in the field of quantitative research on relationship-centered care.

## Introduction

In the veterinary practice, the pet owner plays a key role for achieving a therapy's goals. Adherence to therapeutic measures in human as well as in veterinary medicine has been found to arise from a collaborative relationship with the health professional and a shared decision-making. Therefore, successful communication and relationship-building with the pet owner are essential tools to provide the best care for the patient (1–6).

During decades of research on the physician-patient-relationship, in human medicine and medical sociology, relationship centered care (RCC) was established in accordance to the growing need of equal participation in medical decision making (7–9). While the medicine of the twentieth century has made the biomedical aspects of illness a categorical imperative for medical care, the philosophy of RCC reminds health professionals of the individual and subjective perception of illness as a biopsychosocial phenomenon. According to that, RCC is based on a joint venture between patients and health professionals, driven by mutual respect and a shared understanding of illness as well as shared goals in therapy (10). It offers the opportunity to meet the basic psychological needs that enable (pet owners') motivation and compliance: autonomy, relatedness and competence (11, 12). In accordance, relationship-centered veterinary care is a collaborative relationship between veterinarian and pet owner that is empowered through mutual understanding, balance of power, and the recognition of the pet owners' perspectives and their expertise in the pets' care (13).

Within the philosophy of RCC, self-awareness and self-care as well as respecting the patients' (or a closely related persons') experience of health/illness and the implementation of a shared decision-making are required from any health professional (7). RCC involves a clear and effective communication and the development and maintenance of trustful relationships within the consultation (10, 14). Relationship-centered visits can be characterized as medically functional, informative, responsive, facilitative, and participatory, while the relative importance of each characteristic varies depending on the situation (15, 16). High quality technical skills should be complemented by the use of support, empathy, and concern as well as explicit probes regarding feelings and emotions (16, 17). In veterinary medicine, these general requirements need to be complemented by respecting the animal patient and recognizing its' role in the pet owner's life (13, 18).

Although up to now research on RCC in the veterinary practice remains limited, the available findings suggest, that the concepts of RCC mainly reflect the pet owners' expectations of a “good appointment.” Four non-technical factors seem to be of utmost importance to provide high quality veterinary care and ensure pet owners' as well as veterinarians' satisfaction:

### Information Giving

The ideal of a relationship-centered, participatory approach thrives through the trustful mutual exchange of information (10). For the pet owner, it is necessary to receive intelligible information about the causes and effects of an illness and about possible therapeutic measures. This enables contribution in the medical decision process, helps pet owners to cope with the uncertainty that goes along with illness and fosters hope for the future (19). Therefore, receiving information is the foremost expectation as to the veterinary appointment and showed to be decisive for the pet owners' satisfaction (20–23). Besides its' content, the way of providing information has proven to be highly important to pet owners. They appreciate medical education and information that fit their level of knowledge and interest, while information may preferably be presented in various formats (19, 20, 22).

### Communication

Good communication is a key factor to establish a reliable and trustful relationship between the veterinarian and the pet owner and finally to achieve therapeutic goals (24). The benefits of a communication style with open-ended question could be shown during several studies (14, 18, 21, 25). Nevertheless, veterinarians still tend to communicate in a directive style that reflects a paternalistic role (26). In this, the veterinarians assume that the pet owners' values match their own and dominate the conversation, leaving the pet owners in a passive role (27). Predominately closed questions limit the scope of the dialogue, leaving barely any room to employ empathic statements for relationship building (26).

Research suggests that pet owners prefer veterinarians that communicate using a non-judgmental attitude, speak in an understandable manner without jargon and in a pace that accommodates assimilation. Veterinarians are supposed to ask the right questions, repeat key information, and answer all questions without causing a sense of hurry (20). In addition to verbal communication skills, the ability to listen actively showed to have high impacts on the perceived quality of medical and veterinary consultations (22, 28, 29).

### Empathy

In human medicine, the ability to show empathy and elicit feelings was found to be essential to establish trustful relationships and is therefore essential to RCC (29, 30). In veterinary medicine, qualitative studies on pet owners' expectations suggested that they expect compassion and empathy in communication, respect for their individuality, kindness, and an environment of confidence, in which sufficient time and goodwill is provided to understand the complex medical contexts. This also applies for the opportunity to disclose health problems as well as concerns and worries, to ask all questions without getting the sense of being foolish or stealing the veterinarians' valuable time (20).

### Shared Decision Making

Several relationship-centered models of medical decision making have been developed in the field of human medicinal sociology during the last decades (31). Especially the model of Shared Decision Making (SDM) gained importance in medical care. Several studies showed that its implementation increases success of therapy and patients' compliance, improve their knowledge and perceived control over the disease and reduces anxiety (32–34). SDM allows that evidence-based, constructive, and mutual accepted solutions can be found and both patients and doctors gain respect for their knowledge and abilities. In veterinary medicine, by making shared decisions pet owners become encouraged to take (shared) responsibility for the success of a therapy and questions of preventive health care (35). Several studies on pet owner focus groups and in-depth interviews revealed that pet owners want to be involved in the decision-making process, appreciate to share their personal perspective, desire to be given multiple options for care In contrast, recent findings suggest that the paternalistic approach with a directive communication style in the consultation still seems be heavily integrated into the veterinary identity (26).

Similar to human medical research, various studies already suggest positive effects of the implementation of RCC in the veterinary practice: Adherence to dentistry and surgical recommendations could be improved (4). Creating an unhurried environment during the consultation and spending more time for education and rapport-building with pet owners enhanced pet owners' visit satisfaction (14, 23). In addition, veterinarians with well-trained relationship-centered communication skills perceived pet owners as being less complaining and more personable and trustful (14). Nevertheless, there's some evidence that pet owners leave veterinary appointments with unmet needs, which may be a result of veterinarians' misperception of pet owners' expectations (21, 36). Directive communication, persuasion, and paternalistic behavior still seem to appear and bear the risk to provoke subconscious defensive reactions (psychological reactance) and therefore threatens the relationship as well as therapeutic goals (26, 37).

Though there have been some qualitative approaches to measure the impacts of RCC and especially SDM on relevant outcomes in veterinary care, quantitative approaches remain scarce (4). Therefore, one aim of the study was to explore structural equation modeling (SEM) as an opportunity to build and evaluate models based on quantitative data in this context and test findings within a broader population. Within the SEM, the relationships between influencing latent factors of RCC such as empathic communication, and outcome factors such as fulfillment of pet owners need for information and characteristics of pet owner loyalty were described. Behaviors and habits that are highly associated with the latent factors were identified, as they might be particularly suitable to improve the veterinarian-pet owner-relationship in daily practice.

## Methods

In order to get a broad overview of the pet owners' perception of the RCC efforts in German veterinary practices, a cross-sectional quantitative approach was chosen. A survey on the German pet owners' perception of RCC-related aspects during veterinary appointments was planned and conducted. Preliminary model assumptions were made during the questionnaire development and statistically evaluated using exploratory factor analysis (EFA) and structural equation modeling (SEM) (38).

### Questionnaire Design

For the purpose of questionnaire development, relevant aspects of a relationship-centered veterinary appointment and the pet owners' expectations were identified within the literature. Based on the findings, questionnaire items were created to measure each aspect. In cases where validated questionnaire-items were available from human medical research (35, 39), those were linguistically adapted to veterinary medicine. Items that were included in the model had to be answered on a 6-point Likert-scale.

The questionnaire items were discussed within a team of veterinary researchers and practitioners. In preparation of the SEM the items were explored for possible underlying latent factors by the experts. The results of these considerations were included in a preliminary theoretical model with latent factors and a related set of measurement items for each. During the discussions, a pet owners' need for the consultation of competing health providers (such as homeopaths, naturopaths) was identified to be an outcome/factor of interest and corresponding items were added. All questionnaire items that were used for the SEM (translated to English) and the referring literature as well as the initially proposed latent factors are presented in Table 1.

**Table 1:** Questionnaire items (translated to English) and corresponding references for a survey on German pet owners' perception of communication with their veterinarian.

**Variable code**	**Questionnaire item**	**Reference/generalized findings**	**Factor**
V9	My vet encourages me to describe my pet's health complaints in explicit detail.	PO expect the vet to ask the “right” questions./Patients like to disclose health problems (22).	Active listening
V10	My vet gives me enough time to consider all my questions and answers.	PO expect communication in an unhurried environment/PO wish for communication in a pace that accommodates assimilation (20).	
V3	My vet listens to me with attentive interest.	PO expect to/should be listened to attentively (20, 22, 28).	
V5	My vet asks me easy understandable questions about what has been going on with my pet.	PO expect clear and understandable questions (22).	Verbal communication
V34	I often get the feeling that my vet has not enough time to answer all my questions.	Pet owners worry they may take too much time of the veterinarian (20, 22).	
V8	My vet uses a concise and non-medical language to explain things to me.	PO expect the vet to speak in an understandable language without jargon (20, 22).	
V1	My veterinarian is a likable person.	PO wish to/should be communicated with in a nice way (20).	
V31	I feel uncomfortable to ask questions because my vet might think I did not listen to his/her explanations properly.	PO worry about appearing foolish by asking questions (20).	
V2	My vet sees my pet as an individual with individual needs.	Discussion/Expert review	Empathic behavior
V6	My vet handles my pet with friendly respect.	Discussion/Expert review (14).	
V24	My vet encouraged me to give my opinion and ideas about what might be the cause of my pet's health issues.	PO wish to/should share their perspectives and beliefs (40).	
V4	My vet accepts my personal point of view without giving disrespectful remarks.	PO like communication with a nonjudgmental attitude (20).	
V7	My vet addresses my worries and fears.	PO like to/should be able to disclose concerns (20, 22, 26, 40).	
V33	I am very nervous during a consultation at the vet's.	Discussion/Expert review	
V11	My vet explained to me the pros and cons of each therapeutic option we had.	PO like to/should be provided with information (22, 39).	Shared Decision Making
V25	My vet explained to me the pros and cons of further diagnostic tests.	PO want to participate in decision making (22, 39).	
V14	My vet asked me whether I can implement the therapies in everyday life.	Adapted from PEF Q9 (SDM measurement tool) (39).	
V16	Summarized variable: My vet and I made a clear determination of what has to be done in cases of cure, no cure, or occurrence of undesirable side effects.	Adapted from PEF Q9 (SDM measurement tool) (39).	
V21	My vet explained the results of diagnostic tests (e.g. x-rays, laboratory results) to me in detail.	PO like to/should be provided with information (22, 39).	
V26	My vet and I weighed the different therapeutic options for my pet together.	PO want to participate in decision making (22, 39).	
V17	My vet and I made a joint decision about which therapy option to choose.	PO wand to participate in decision making (22).	
V35	In general I wish my vet would provide me more information about my pet's health.	PO like to/should be provided with information (20, 22).	Information Giving
V12	My vet asked me what I already knew about my pet's health issue.	PO like to be asked for previous experience.	
V13	My vet asked me how much more information I would like to get about my pet's health issue.	Evaluate information preference (20).	
V15	My vet explained to me in detail which undesirable side-effects may result from the medication.	PO like to/should be provided with information (22).	
V18	My vet explained to me the effects of each prescribed drug.	PO like to/should be provided with information (22).	
V19	My vet encouraged me to learn more about my pet's health.	Activate PO as a goal of SDM	
V36	My vet handed me a detailed medication plan.	PO like to/should be provided with information.	
V20	My vet explained to me precisely how the therapeutic measures need to be done.	PO like to/should be provided with information.	
V23	My vet showed me in detail how I should apply the drugs correctly (e.g. how I make sure my cat is taking its pill)	PO like /should be provided with information.	
V30	I wish my vet would be more open to alternative/complementary treatment options.	PO like to be given different options of care (22).	
V32	In general many questions occur only after I arrived at home/left the veterinarian.	Discussion/Expert review.	
V27	Before a treatment or examination was done, my vet informed me about the anticipated costs.	PO like to discuss out-of-pocket costs (21).	
V37	I always followed the instructions my vet gave me.	Discussion/Expert review.	Pet owners' need for alternatives
V22	I was satisfied with the decisions that have been made.	Discussion/Expert review.	
V28	I already consulted an alternative health provider or homeopath because of dissatisfaction with my vet's care.	Discussion/Expert review.	
V29	I already consulted an animals' physiotherapist or osteopath because of dissatisfaction with my vet's care.	Discussion/Expert review.	

*Table includes variable code and initially proposed latent factors for a preliminary theoretical model. Preliminary model assumptions were verified by means of exploratory factor analysis and structural equation modeling. PO, Pet owner*.

In addition to the items used for modeling, general questions were added to the questionnaire. They referred to type and number of pets, cause, and number of the latest veterinary appointments, characteristics of the actual veterinary practice, the decision-making preference and demographic data. At the end of the questionnaire participants were able to leave individual comments in a voluntary comment field.

The questionnaire was validated using a three-step pretesting process with expert reviews, cognitive pretesting, and standard pretesting. In the first step, the questionnaire draft was sent to six interdisciplinary experts (veterinarians, psychologists, social scientists). They were asked to check all items for relevance within the context of veterinarian-pet owner communication, RCC, and SDM as well as with regard to the methodology of SEM-building. Expert review was followed by a cognitive pretest with 12 participants. Within the cognitive pretest, all items were checked for validity using methods of paraphrasing and thinking aloud (41). Items or phrasings that showed to be misleading were revised or exchanged after the first six cognitive pretests. The revised questionnaire showed good validity within the remaining six cognitive pretests and was rated to be easy to understand by the pretesters. This could be confirmed in a final quantitative pretest with 26 participants (42).

The final questionnaire comprised 58 items including general questions and demographic data.

### Data Collection

A nationwide survey among pet owners was conducted from the 15th of August 2016 until the 30th of October 2016. Eligible to participate were people that owned at least one companion animal and visited a veterinarian practice in terms of medical check-ups, illnesses or operations in the last 2 years. Data collection, storage, and processing was done in accordance with the current German data protection laws. Each participant was adequately informed of the aims, methods, and scope of the survey. Informed consent had to be given actively before the survey could be started. Participation was voluntary. Data collection was anonymous and no personal nor other sensible data were collected. The survey could be terminated at any point. Therefore, no approval by an ethics committee was required as per the local legislation.

To acquire a large number of participating pet owners, the questionnaire was provided online (LimeSurvey, open-source, hosted on university servers) as well as paper-based. A professional information website (www.fokustiergesundheit.de) with an external link to the survey page was developed in cooperation with a web designer and promoted in 281 local and nationwide pet-associated Facebook groups after administrators were asked for permission. In addition, a project Facebook page was created and shared. German Equestrian Association (FN) supported the distribution by Facebook postings. Twenty-seven horse stables, equestrian shops, and dog trainers supported the study as well and received information flyers and posters. Overall 100 questionnaire hardcopies were sent to all parts of the country. Nonetheless the study population cannot be regarded as representative due to the convenient sampling strategy. From 1,270 completed questionnaires, 1,745 have been answered online, while 25 participants used the hardcopy version.

### Data Analysis

Data were extracted from LimeSurvey and the hardcopy questionnaires, stored in a Microsoft Excel® 2016 file and statistically analyzed using SAS version 9.4 (SAS Institute Inc., Cary, NC, US).

None of the data sets had to be deleted due to non-plausible data such as unrealistic values of age (<15 years >99 years) or numbers of companion animals owned after data inspection.

Items used for modeling were visually checked for normality (histograms and Q-Q-plots) and by Kolmogorov-Smirnov tests. Normality was not given for all items. Descriptives were calculated for all relevant variables including mean, min, max, standard deviation, kurtosis, and skewness.

Missing data imputation was performed in terms of deterministic regression imputation in cases of occasional missing answers in preparation for multivariate data analysis.

#### Exploratory Factor Analysis

Because of the limitation of assured previous knowledge in the field of relationship-centered communication in German veterinary medicine, an explorative approach of data analysis was chosen. In a first step data were analyzed using Exploratory Factor Analysis (EFA) to characterize the underlying constructs and confirm theoretical model assumptions.

Estimations were done using the proc calis statement with squared multiple correlations as prior communality estimates, followed by a promax (oblique) rotation. Because normality was not valid for all items, the unweighted least squares (ULS) method was used.

Scree test and Eigenvalues were used to select the suggested number of factors (43). Cronbach's alpha was calculated to assess reliability of the factor constructs. α-values >0.70 were considered good, α > 0.80 were considered to be ideal (44). The results of the EFA were compared to the preliminary model.

#### Structural Equation Model

**Figure 1:**
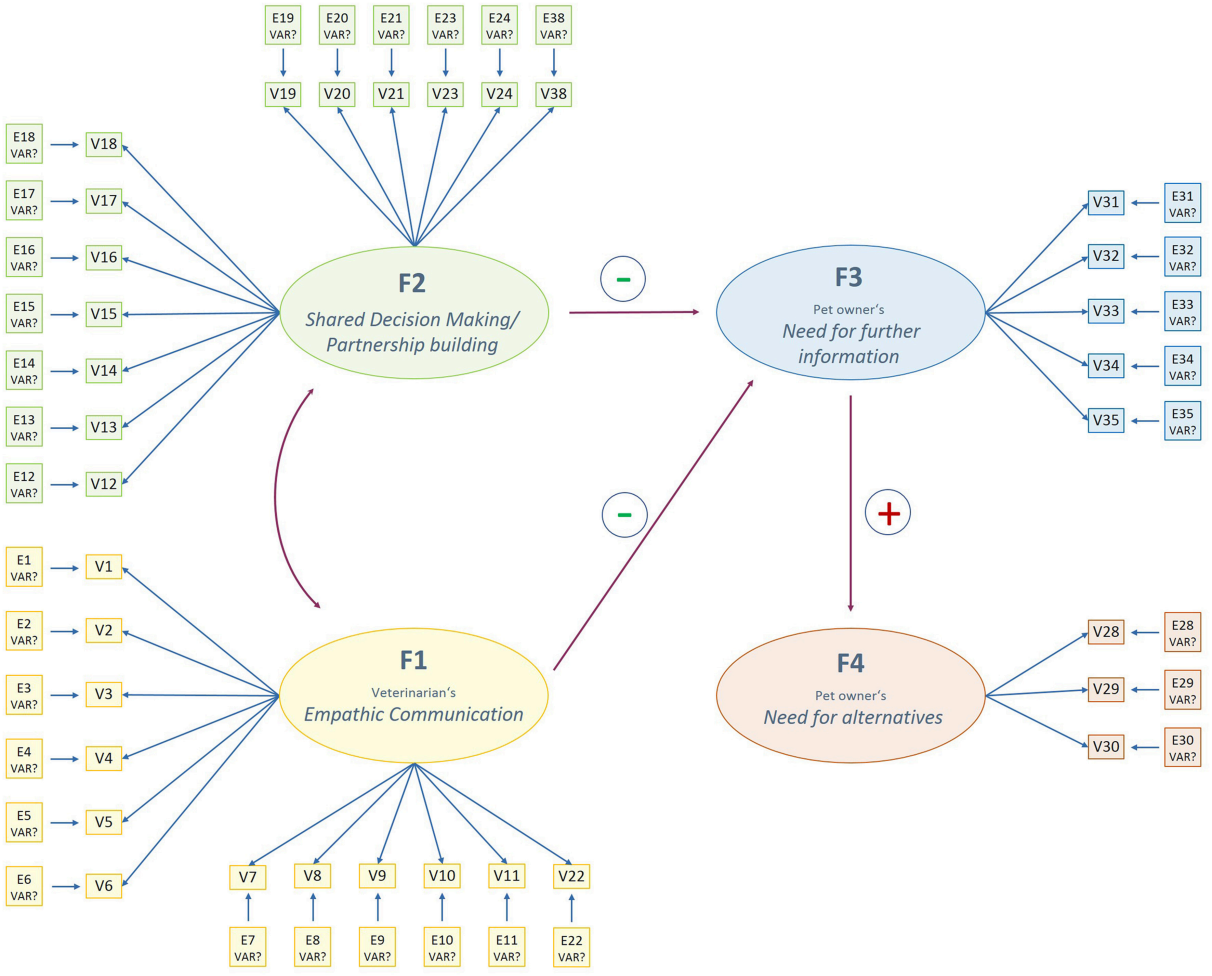
Preliminary structural equation model describing latent factors of pet owners' perception of communication with their veterinarian. Directional single-headed arrows describe hypothesized directional influences. The curved double-headed arrow hypothesizes a bilateral correlation between factors. F, latent factor. V, measurement item. E, Error term.

Based on the findings of the EFA a structural equation model (SEM) with directional paths between latent factors was created (Figure 1). The aim of the SEM was to describe the nature of the relationship between the latent (= not directly measurable) factors that were identified from literature research and exploratory factor analysis, and the measured indicator variables that were suggested to measure those latent factors.

Latent variables were labeled with the letter “F.” As recommended, each latent factor was measured by at least three indicator variables labeled by the letter “V.” Error terms were named using the letter “E” (45).

The model was estimated using the unweighted least square method in the CALIS procedure (46). For each latent construct, the estimate of the factor loading of the variable with the highest loading on this factor in EFA was fixed to one in the linear equation.

Root Mean Square Error of Approximation (RMSEA), Standardized Root Mean Square Residual (SRMR) and Bentler Comparative Fit Index (CFI) were used to assess goodness-of-fit of the model. For a model fit to be acceptable, the upper level of the confidence interval of RMSEA and the value of SRMR should not exceed 0.09, and CFI should be >0.90 (38, 47).

Factor loadings as well as *t*-values and indicator reliability (*R*^2^) for each variable and the composite reliability for the respective factor were calculated. Variance extracted estimates for each indicator variable were analyzed to measure the amount of variance captured by each construct. Significant *p*-values indicate that factor loadings differ significantly from zero in large-sample *t*-test (*p* < 0.01). Indicator reliability above 0.39 was considered as ideal. Composite reliability analogous to Cronbach's' Alpha is said to be good if α > 0.70 and ideal if α >0.80 (38). Variance extracted estimates were considered for each latent construct as well for all constructs combined by computing the arithmetic mean and said to be good in excess of 0.50 (48).

Lagrange Multiplier and Wald test were used to check whether deleting a path or covariance in the model would significantly improve the model fit (38).

## Results

### Sample

In total, 1,434 online and 25 paper-based questionnaires were returned. Of those, 189 online questionnaires were classified as incomplete (<40% of the model-related items completed) and therefore excluded from the study. The remaining 1,270 participants had a median age of 38 years, 88.4% were female. 55.6% owned only small companion animals, 7.6% were horse owners, and 36.9% held both horses and small companion animals. Most of the participants owned animals for more than 10 years (73.5%), while 11.7% had six to ten, 9.6% two to five, and 5.1% <2 years' experience in being a pet owner. 74.2% of the participants preferred SDM during a consultation, while only 14.6% preferred a paternalistic, 11.3% a professional-as-agent model of medical decision making.

The 40 variables relevant for EFA/SEM were selected from the 58 overall variables. Descriptive statistics of all 38 items considered for structural equation modeling are summarized in Table 2.

**Table 2:** Descriptive statistics of items used in the final structural equation model with four latent factors to describe pet owners' perception of the communication with their veterinarian.

**Descriptive analysis of items used in the final model**										
**Variable**	***N***	**Mean**	**Min**	**Max**	**Std dev**	**Std error**	**Lower 95% CL for mean**	**Upper 95% CL for mean**	**Kurtosis**	**Skewness**
V1	1,270	5.41	1.00	6.09	1.03	0.03	5.36	5.47	4.52	−2.10
V2	1,270	5.30	1.00	6.00	1.07	0.03	5.24	5.36	3.50	−1.86
V3	1,270	4.96	1.00	6.00	1.28	0.04	4.89	5.03	1.03	−1.29
V4	1,270	4.84	1.00	7.52	1.32	0.04	4.78	4.92	0.90	−1.19
V5	1,270	5.21	1.00	7.86	1.20	0.03	5.14	5.27	2.19	−1.56
V6	1270	5.65	1.00	6.00	0.81	0.02	5.60	5.69	10.66	−2.30
V7	1,270	5.02	1.00	6.94	1.30	0.04	4.94	5.09	1.27	−1.38
V8	1,270	5.17	1.00	7.07	1.14	0.03	5.11	5.23	2.45	−1.62
V9	1,270	5.02	0.97	8.28	1.31	0.04	4.95	5.10	1.14	−1.31
V10	1,270	4.87	0.90	7.97	1.38	0.04	4.79	4.94	0.32	−1.06
V11	1,270	4.30	0.66	8.62	1.65	0.05	4.21	4.40	−0.81	−0.55
V12	1,270	3.60	−0.34	7.99	1.69	0.05	3.51	3.69	−1.09	−0.03
V13	1,270	3.41	−0.05	7.47	1.72	0.05	3.32	3.51	−1.19	0.07
V14	1,270	3.21	−0.60	7.48	1.81	0.05	3.12	3.31	−1.25	0.23
V15	1,270	3.67	0.37	7.67	1.68	0.05	3.58	3.76	−1.10	−0.07
V16	1,270	4.43	1.00	7.41	1.39	0.04	4.35	4.51	−0.39	−0.65
V17	1,270	4.30	0.32	8.41	1.77	0.05	4.20	4.40	−0.93	−0.55
V18	1,270	4.15	−0.34	7.80	1.55	0.04	4.07	4.24	−0.62	−0.50
V19	1,270	4.10	1.00	9.13	1.70	0.05	4.01	4.20	−1.02	−0.39
V20	1,270	4.62	0.86	7.76	1.50	0.04	4.54	4.70	−0.06	−0.90
V21	1,270	5.00	1.00	9.04	1.34	0.04	4.93	5.07	0.76	−1.10
V22	1,270	5.00	1.00	7.32	1.29	0.04	4.93	5.07	1.21	−1.32
V23	1,270	4.67	−0.36	7.72	1.52	0.04	4.59	4.76	−0.03	−0.95
V24	1,270	4.21	0.20	8.40	1.66	0.05	4.12	4.30	−0.89	−0.49
V25	1,270	4.57	0.92	8.04	1.51	0.04	4.49	4.65	−0.26	−0.79
V26	1,270	4.31	0.19	8.68	1.73	0.05	4.22	4.41	−0.84	−0.58
V27	1,270	3.17	−1.52	6.62	1.85	0.05	3.07	3.28	−1.33	0.26
V28	1,270	2.50	−2.01	7.63	1.94	0.05	2.39	2.61	−0.82	0.81
V29	1,270	2.50	−2.24	7.76	1.93	0.05	2.40	2.61	−0.84	0.81
V30	1,270	3.26	−0.79	7.67	1.83	0.05	3.16	3.37	−1.33	0.16
V31	1,270	1.95	1.00	6.00	1.26	0.04	1.88	2.02	0.74	1.23
V32	1,270	3.03	1.00	6.00	1.46	0.04	2.95	3.11	−0.78	0.33
V33	1,270	3.02	1.00	6.00	1.59	0.04	2.93	3.12	−0.93	0.36
V34	1,270	2.57	1.00	6.00	1.56	0.04	2.48	2.65	−0.60	0.70
V35	1,270	2.80	1.00	6.29	1.66	0.05	2.71	2.89	−0.96	0.52
V36	1,270	5.38	1.00	8.52	1.17	0.03	5.31	5.44	3.54	−1.81
V37	1,270	5.39	1.00	7.89	0.98	0.03	5.33	5.44	4.27	−1.85
V38	1,270	4.40	1.00	7.85	1.51	0.04	4.31	4.48	−0.65	−0.62

*Imputed data set*.

### Exploratory Factor Model

In contrast to the preliminary model, EFM's scree test, and Eigenvalues suggested only four factors (instead of six). As the previously considered factors veterinarians' *Verbal communication* and veterinarians' *Active listening* and veterinarians' *Empathic behavior* were theoretically closely related, three factors were collapsed to one overall factor labeled *Empathic Communication* factor. The remaining model was labeled “Revised model 1.”

In interpreting the rotated factor pattern, an item was said to load on a given factor if the factor loading was at least 0.39 for that factor and <0.39 for another.

Applying these criteria, two items (V36, V37) were no longer considered. The remaining questionnaire items and the corresponding factor loadings are presented in Table 3. Latent factors were labeled F1 (*Empathic Communication* factor), F2 (*Partnership-Building* factor), F3 (*Need for further Information* factor), and F4 (*Need for Alternatives* factor). Eleven items were found to load on the *Empathic Communication* factor, 15 items were found to load on the *Partnership-Building* factor, and five items loaded on the *Need for further Information* factor. Finally, three items remained loading on the *Need for Alternatives* factor.

**Table 3:** Standardized regressions coefficients in the rotated factor pattern after exploratory factor analysis (EFA).

**Rotated factor pattern (standardized regression coefficients)**				
	**Factor1**	**Factor2**	**Factor3**	**Factor4**
V1	95 *	−4	4	4
V2	81 *	6	6	−2
V3	74 *	12	−10	−3
V4	61 *	16	−8	2
V5	56 *	22	3	2
V6	89 *	−16	6	3
V7	75 *	16	−4	1
V8	68 *	7	−7	−6
V9	53 *	31	7	3
V10	49 *	31	−12	3
V12	−9	86 *	−5	8
V13	−9	92 *	−5	6
V14	−8	84 *	7	−4
V15	−4	82 *	7	−7
V16	24	61 *	8	−9
V17	17	71 *	−5	8
V18	14	67 *	6	−7
V19	19	63 *	−1	−2
V20	21	60 *	12	−9
V21	31	50 *	−3	7
V22	44 *	36	4	−13
V23	27	50 *	14	−5
V24	25	61 *	−9	10
V28	−1	3	−5	89 *
V29	2	1	−1	80 *
V30	4	−23	29	39 *
V31	−3	9	81 *	−5
V32	3	−11	68 *	3
V33	7	14	53 *	−4
V34	−24	−10	46 *	9
V35	−16	−36	42 *	12
V38	19	77 *	−2	6

*Results based on 1,270 completed questionnaires measuring pet owners' perception of communication with their veterinarian*.

Printed values are multiplied by 100 and rounded to the nearest integer. An item was said to load on a factor if the factor loading was >0.39 for one factor (flagged with ^*^) and < 0.39 for the others.

Cronbach's test for reliability showed acceptable to high standardized α = 0.94 for *Empathic Communication* factor, α = 0.95 for *Partnership-Building* factor, α = 0.73 for *Need for further Information* factor, and α = 0.79 for *Need for Alternatives* factor. Due to the high intercorrelations among the measures in Cronbach's alpha and also close substantive relationship, V11, V25, and V27 were summarized in a new variable (V38) calculated by using the arithmetic mean over the three variables. Recalculation still showed good values of α = 0.95 for *Empathic Communication* factor, α = 0.95 for *Partnership-Building* factor, α = 0.80 for *Need for further Information* factor, and α = 0.79 for *Need for Alternatives* factor.

### Structural Equation Model

Goodness-of-fit indices showed partly unsatisfactory model fit (RMSEA = 0.0870 (confidence interval 0.0848-−0.0892), SRMR = 0.0693, and CFI = 0.84). The Wald Test suggested to delete the directional path between F1 and F4 as a way to significantly improve the model fit. Because of the conceptual argumentation that empathy and a partnership-based SDM were depending each other, this change could be justified and therefore was tested in a revised model named “Revised model 2.”

**Figure 2:**
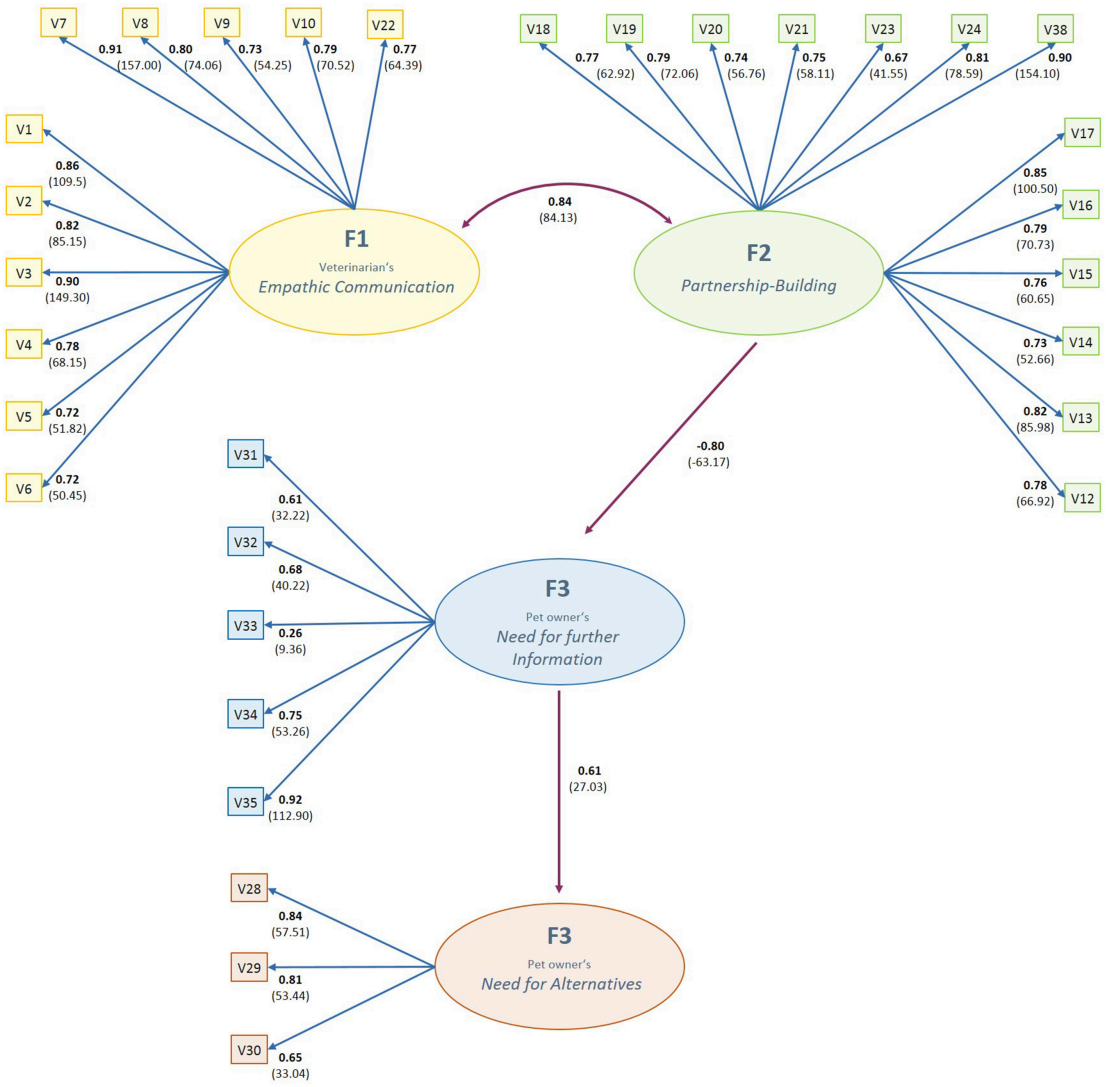
Final structural equation model describing latent factors of pet owner's perception of communication with their veterination. Association of latent factors and items described by path coefficients in bold and t-values in parentheses. F, latent factor; V, measurement item. Result based on 1,270 questionnaire responds.

The revised model with the deleted path is shown in Figure 2. Goodness-of-fit indices of the revised model showed slightly improved model fit with RMSEA <0.09 (0.0848 confidence interval 0.0829−0.0866), SRMR <0.08 (0.0672) and CFI quite close to 0.90 (0.87).

Further recommendations given by the Lagrange Multiplier/Wald test could not be justified on theoretical basis, so the model could not be further improved with the given data/item structure.

Standardized factor loadings as well as *t*-values and indicator reliability (*R*^2^) for each variable and the composite reliability for the respective factor are presented in Figure 2 and Table 4. Standardized loadings showed a range from 0.26 (V33) to 0.92 (V35) with all being statistically significant at *p* < 0.01 and none of them being trivial (<0.5) except V33. Indicator reliability showed to be ideal (>0.39) for most of the items except V31 and V33. Composite reliability was good to excellent for all factors with coefficients above 0.70 (marked with ^b^).

**Table 4:** Properties of the structural equation model with four latent factors describing pet owners' perception of communication with their veterinarian.

**Properties of the revised model**				
**Constructs and indicators**	**Standardized loading**	***t***^**a**^	**Reliability**	**Variance extracted estimate**
Empathic communication [F1]			0.95^b^	0.64
V1	0.86	109.50	0.74	
V2	0.82	85.15	0.68	
V3	0.90	149.30	0.81	
V4	0.78	68.15	0.61	
V5	0.72	51.82	0.52	
V6	0.72	50.45	0.51	
V7	0.91	157.00	0.82	
V8	0.80	74.06	0.64	
V9	0.73	54.25	0.54	
V10	0.79	70.52	0.62	
V22	0.77	64.39	0.59	
SDM/Partnership [F2]			0.95^b^	0.61
V12	0.78	66.92	0.61	
V13	0.82	85.98	0.68	
V14	0.73	52.66	0.53	
V15	0.76	60.65	0.57	
V16	0.79	70.73	0.62	
V17	0.85	100.50	0.72	
V18	0.77	62.92	0.59	
V19	0.79	72.06	0.63	
V20	0.74	56.76	0.55	
V21	0.75	58.11	0.56	
V23	0.67	41.55	0.45	
V24	0.81	78.59	0.65	
V38	0.90	154.10	0.81	
Need for further information [F3]			0.79^b^	0.46
V31	0.61	32.22	0.38	
V32	0.68	40.22	0.46	
V33	0.26	9.36	0.07	
V34	0.75	53.26	0.56	
V35	0.92	112.90	0.84	
Need for alternatives [F4]			0.81^b^	0.59
V28	0.84	57.51	0.70	
V29	0.81	53.44	0.65	
V30	0.65	33.04	0.42	

*Standardized factor loadings describe the relationships between measurement item and underlying latent factor. Variance extracted estimates measure the amount of variance captured by each latent construct. Results based on 1,270 questionnaire responds*.

*Calculations were done using SAS version 9.4*.

a *All t tests were significant at p < 0.01*.

b *Denotes composite reliability*.

Variance extracted estimates were good for F1, F2, and F3 and acceptable for F4 with a good overall value of 0.58 for all constructs combined. These findings support the assumption of the validity and reliability of the constructs and their indicator variables. The revised was therefore named “Final Model” and retained for discussion.

#### Empathic Communication (F1)

The *Empathic Communication* factor was mostly associated with the veterinarians' efforts to address the pet owners' worries and fears (V7: ß = 0.91), followed by the veterinarians' ability to listen to the pet owner attentively and with interest (V3: ß = 0.90). A likable manner of the veterinarian (V1: ß = 0.86), taking the individual characteristics of the pet into consideration (V2: ß = 0.82) and using a concise non-medical language (V8: ß = 0.80) also showed high associations with this factor. The aspects of giving the pet owner enough time for consideration (V10: ß = 0.79) as well as respecting and accepting the pet owners' point of view without giving (verbal or non-verbal) disrespectful remarks (V4: ß = 0.78) had a less but still remarkably high loading on the Empathy factor. The pet owner's satisfaction with the decision made was also remarkably directly associated with this factor (V22: ß = 0.77).

#### Partnership-Building (F2)

The *Partnership-Building* factor was mostly associated with discussing pros and cons of diagnostic and therapeutic options (V38, ß = 0.90), making a shared decision in choosing the therapy (V17: ß = 0.85), asking the pet owner for his or her need for further information (V13: ß = 0.82) and asking the pet owner for his or her opinion/ideas, what the cause of the illness might be (V24: ß = 0.81). Further highly associated items were the clear determination of what has to be done in cases of cure, no cure, deterioration in the state of health or occurrence of undesirable side effects (V16: ß = 0.79), encouraging the pet owner to learn more about his pets' health issues (V19: ß = 0.79), asking for the pet owners' previous knowledge about the actual question of health (V12: ß = 0.78) and providing information about effects (V18: ß = 0.76) and undesirable side effects of the medication (V15: ß = 0.76).

#### Need for Further Information (F3)

The factor *Need for further Information* was mostly associated with an unmet wish for more medical information (V35, ß = 0.92), a feeling that the veterinarian lacks time to answer (further) questions (V34, ß = 0.75), and the pet owners being uncomfortable in asking more questions because the veterinarian might think they did not listen properly (V31: ß = 0.61). The pet owners' feelings of being nervous during a consultation were considerably less associated with this factor (V33, ß = 0.26).

#### Need for Alternatives (F4)

The pet owner's desire to explore alternative ways of medical care was highly associated with their considerations to consult physiotherapists/osteopaths (V28: ß = 0.84) or alternative/homeopathic non-medical practitioners due to dissatisfaction with the vet's care (V28; ß = 0.84) and their unfulfilled wish for more complementary therapy options offered by the veterinarian (V30, ß = 0.60).

#### Overall Model

Standardized results for covariance among factors F1 and F2 showed a high correlation between a veterinarians' *Empathic Communication* and the extent of *Partnership-Building* due to participation during the decision-making process (0.82). The standardized path coefficients connecting F2 with F3 showed a highly negative factor loading of PF2F3 = −0.80 whereas the path connecting F3 with F4 showed a loading of PF3F4 = 0.61. Consideration of the standardized results for variances of exogenous variables showed that only 36% of the variance in *Need for further Information* (F4) had to be accounted for by the exogenous disturbance term (D3 = 0.36 with *t* = 17.73), 63% of the variance in *Need for Alternatives* (F3) had to be accounted for by the exogenous disturbance term (D4 = 0.63 with *t* = 23.35). In return, 64% of the variance in the *Need for further Information* factor can be accounted for by veterinarians' *Empathic Communication* and *Partnership-Building* factors F1 and F2 whereas 37% of the variance in the *Need for Alternatives* factor can be accounted for by the *Need for further Information* factor.

## Discussion

The relationship between doctors and people that are facing illness is one of the most complex social interactions. Patients and closely related persons have to interact with a rather unfamiliar person (the doctor) in a non-equal position and trust him or her to handle highly sensitive issues of vital importance. Decisions need to be made within emotional situations of fear, uncertainty, distress, and weakness (49). Differences in philosophies of life and health, low levels of health literacy on the patients' side as well as possible deficits in communication skills and empathy on the professionals' side increase the levels of complexity in this situation (26, 50, 51). In todays' enlightened western society, the pet often takes the role of a beloved companion, is part of the family and is sometimes even given a child-like status (52–54). In consequence, the currents observed in the physician-patient-relationship reflect on the veterinarian-pet owner-relationship as well: Pressure increases for veterinarians to provide high quality, efficient care—often within financial limitations—, and communication during consultations has to meet nearly as high requirements as in human medicine (55, 56). In the face of growing concerns about the well-being of veterinarians, appropriate measures to ensure veterinarians' work, and life satisfaction are urgent (57–61). Over the last decades, RCC has turned out to provide a promising approach to deal with the changing expectations and provide high-quality (veterinary) health care (4, 27, 62). Several studies with mainly qualitative designs suggested, that the implementation of components of RCC (e.g., open-minded communication, providing information, respect the pet owners' point of view) improves not only pet owners', but also veterinarians' satisfaction (14, 62, 63).

First aim of this study was to explore structural equation modeling (SEM) as an opportunity to build and evaluate models based on quantitative survey data in the context of relationship-centered veterinary care. Further, the relationships between influencing latent factors of RCC and relevant outcome variables should be described. For practical implication, behaviors and habits should be identified that are highly associated with the latent factors and therefore may be particularly suitable to improve the outcomes in daily practice.

The approach of structural equation modeling proved to be a valuable way to statistically analyze the complex latent structures that make up a relationship-centered appointment. Due to exploratory factor analysis, the latent factors *SDM/Partnership-Building, Empathic Communication, Pet owners' Need for further Information* and *Pet owners' Need for Alternatives* could be identified. These were used for structural equation modeling; the results are discussed in the following sections.

### Empathic Communication and Partnership-Building

To implement RCC in veterinary care, building up a trustful partnership (“joint venture”) is essential. This partnership builds on mutual respect for perspectives, interest, expertise as well as a mutual exchange of information and a shared decision-making (27). Previous studies suggest, that these requirements meet the pet owners' expectations of good veterinary care in large parts (19, 20, 22).

Within this study, the principles of SDM were supported by 74.2% of the participants. 14.6% preferred a paternalistic model in which the veterinarians take the decisions and merely inform the pet owners. 11.3% of the respondents favored a professional-as-agent model of medical decision making, in which the veterinarian provided all relevant information to the pet owner who then takes the decision autonomously. Therefore, the percentage of veterinarians feeling comfortable with the SDM model is remarkably higher than in human medicine where in average only 55.5% prefer SDM (64). This may refer to the common practice of pet owners having to pay directly for veterinary medical services: Diagnostic and therapeutic options need to be chosen on basis of financial considerations in most of the cases. This causes the general necessity to discuss at least the basic decision options with the pet owner as a paying person (21).

In contrast to that, many pet owners' critically commented in the voluntary field at the end of the study's questionnaire. The statements showed that the implementation of a partnership-based approach still seems not to be common practice in Germany. A recent study of Bard et al. (26) therefore suggest that veterinarians may already be motivated to create an environment that meets the pet owners' needs for empathy and collaboration, but still tend to act in a paternalistic and persuasive way (26). This discrepancy might be caused by lack of conviction and/or lack of competences and could initiate future research.

Acceptance of the concepts and philosophy of RCC and SDM will be required for a successful implementation of relationship-centered veterinary care with its advantages (10). Veterinarians need to accept that within his or her abilities, every pet owner like every human patient is able to take part in a decision provided that information are accessible and easy to understand (65). Therefore, veterinarian practitioners should pursue their medical educational tasks and encourage pet owners to learn more about their pet's health. Remarkably, enhancing the pet owners' comprehension seems not to lead to significantly longer appointment times, but increases the efficiency of the consultation (20). This may result in an improved animal health literacy and improved animal healthcare provision. Moreover, due to today's opportunities of easy (digital) information-sharing, a passive but demanding “consumers-self-perception” of pet owners should no longer be accepted. Instead, pet owners should be encouraged to take the responsibility for their pets' health which includes the willingness to acquire knowledge about health and (preventive) health care. Further investigations among veterinarians should be done to get deeper insights in explicit motives of using or ignoring concepts of RCC to find practicable solutions.

Well-trained veterinarian interpersonal and communication skills are necessary to meet the requirements of RCC. Within a study on the effects of an aimed educational program for veterinarians, training showed to enhance overall visit satisfaction of both pet owners, and veterinarians. After the intervention veterinarians perceived “their” pet owners as less complaining but more personable and trusting, whereas pet owners felt more involved and respected (14). Within this study, basic concepts of good communication skills that are recommended in the Calgary Cambridge Guide for medical interviews, showed to be highly associated with the *Empathic Communication* factor while having excellent values of reliability. Addressing the pet owners' worries and fears (V7: ß = 0.91) as one of the most deeply humanistic duties of the medical profession (66) and active listening (V3: ß = 0.90) as a “number one expectation” on a good doctor (67) showed their potential importance for veterinary medicine by highest factor loadings, too. Taking the individual characteristics of the pet into consideration (V2: ß = 0.82) had an even larger factor loading than respecting and accepting the pet owners' perspectives without giving (verbal or non-verbal) disrespectful remarks (V4: ß = 0.78). This emphasizes the importance of not only focusing on the pet owner but also on the pet as an individual. In addition to the benefits for the pet owners' feeling and the veterinarian-pet owner-relationship, empathic acting has shown to raise doctors' as well as veterinarians' work satisfaction and correlates with decreased burnout (14, 62, 68).

Being likable (V1: ß = 0.86) and speaking in a concise non-medical language (V8: ß = 0.80) turned out to be promising tools in strengthening a positive relationship due to compassionate acting. Similar findings have been seen in several studies that highlight the importance of relationship-building as vital to the success of every appointment and found empathy to be a central key for building good relationships. (40, 69). The high correlation between the *Partnership-Building* and *Empathic Communication* factor in the model confirmed these considerations. In this context, it is quite important to pay attention to a precise understanding of the notion of empathy. In a general sense, to be empathic is to put oneself in someone else's shoes or to see a problem from another person's position (17). More precisely, there's various forms of empathy and unfortunately definitions are not consistent. As described in Jeffrey (70), within health professionals empathy often is understood in a *self-orientated* way, which is mentally exhausting. Therefore, empathy should rather be practiced in an *other-orientated* way. (71) This allows the veterinarian to explore the situation from the pet owner's point of view and therefore supports the implication of a RCC approach (70).

### Need for Further Information

Giving patients the desired amount of information through clear and thorough explanations may help pet owners to feel more hopeful and to manage uncertainty by gaining a greater sense of control (20). In addition, analogous to human medicine this may lead to increased satisfaction (72). Therefore, meeting the pet owners' expectations of being well-informed also seems to be a desirable goal for veterinarians.

In our study, the factor *Need for further Information* was mostly associated with an unmet wish for more medical information (V35, ß = 0.92) and a feeling that the veterinarian lacks time to answer all questions (V34, ß = 0.75). Further, if pet owners felt uncomfortable to ask all their questions, because they were afraid to be regarded as inattentive listeners, they tend to have a higher need for further information, too (V31: ß = 0.61).

This supports the hypothesis that providing information, creating a calm atmosphere during the consultation without giving verbal or non-verbal signals of time pressure as well as inviting pet owners to ask further questions decreases the *Need for further Information*. The possible influences of providing e.g., written information or recommending other information sources were not addressed in the questionnaire but could be an interesting option. Especially the possible benefits of implementing evidence-based decision aids (73) in veterinary medicine could be interesting topics for future research.

In the century of digital revolution, a positive influence on pet owners' information-seeking behaviors is rapidly gaining importance. The internet became a source of health related information for the majority of pet owners that seek for (further) information (74). Internet-sources in many cases still provide misleading or inaccurate health information and pet owners often lack the required levels of health literacy to filter valid and invalid information (51, 75, 76). An uncritical use of internet-sources for veterinary information therefore poses the risk of inducing fear, false ideas and expectations on the pet owners' side. Additionally, the use of invalid internet sources may cause dissatisfaction in medical professionals and has also the potential to impair the trust between veterinarian and pet owner (9). Therefore, it should become a goal for the veterinary profession to satisfy the need for information and encourage pet owners to develop higher levels of health literacy by providing appropriate information materials. Thus, not only the pet owners take advantage from improved information giving as they feel understood and confident to support a good health care for their pets: Also veterinarians may gain from being a good partner in medical decisions in economic ways (77) as well as in dealing with pet owners interpersonally.

Remarkably, the pet owners' feelings of being nervous was less associated with the *Need for further Information* (V33, ß = 0.26). Distress has proven to have a negative impact on cognitive performance during a consultation in previous studies (72, 78). This contributes to our conclusion that besides the factor of longing for more information but feeling uncomfortable to ask questions, another independent factor was missing in the model that represented anxiety or uncertainty [supported by Kuhltau (79)].

### Need for Alternatives

Achieving loyalty is a matter of significant financial impact on most businesses and therefore one of the primary marketing goals (80). In general, customers tend to measure “service quality” on their subjective emotional experience, rather than on the actual more objective quality (81). Increasing numbers of alternative health providers (such as homeopaths, naturopaths etc.) in Germany appear to suggest that pet owners gladly accept such opportunities to achieve medical advice beyond the veterinary practice (82). Therefore, a pet owners' need for alternative health providers was identified to be an outcome of interest during expert discussions.

Recent research demonstrated that loyalty of pet owners is especially influenced by an affective relationship with the veterinarian and their satisfaction with veterinarian communication (both aspects of RCC). In turn, loyalty predicted other outcomes of interest such as a pet-owner's perception of the cost and value of veterinary services (77). This leads to the conclusion that RCC may be beneficial to pet owners' loyalty. Within this study, the pet owners' needs to exploit alternative ways of medical care was highly associated with their considerations to consult physiotherapists or osteopaths (V28: ß = 0.84) or complementary non-medical practitioners due to dissatisfaction with the veterinarian (V28; ß = 0.84). Besides it was clearly associated with an unfulfilled wish for more complementary therapy options offered by the veterinarian (V30, ß = 0.60). A large part (37%) of this factor's variance could be accounted for by the latent factors of *Empathic Communication, Partnership Building*, and fulfillment of the *Need for Information* within the model. This supports earlier findings that satisfaction in medical decisions is subject to the amount of information given by providers and a relationship- and pet-centered care with respect for the pet owner's perspective and opinion (13, 83).

Since a large part of the factor's variance remained unaccounted for in the model it can be hypothesized, again, that there may be more latent factors that have not adequately been taken into consideration so far. In addition to the already mentioned *Anxiety/Uncertainty* factor it seems possible that—probably connected with this factor—outcomes of earlier consultations or even traumatic experiences as well as the pet owner's perception of the *Veterinarian's technical skills* could be essential predicting factors. Further investigations should rethink corresponding extended models and consider, whether these changes will give the opportunity to improve the model fit and provide more reliable data for the factor F4.

### Conclusion and Outlook

#### Limitations

Although the sample size of 1,270 valid answers was sufficiently large to build the SEM model, the results cannot be regarded as representative. The convenient sampling strategy used did not provide tools to control that all types of pet owners were represented evenly. As the sampling strategy was focused on social media users, an overrepresentation of younger female people with high affinity to web based information sources is probable. Potentially people working on a part-time position were more disposed to participate in the questionnaire while elderly people with restricted use of the internet could not be reached very well. For the study was conducted in Germany, findings may only be applied to countries with similar values referring to the human-animal bond and a need for participation in veterinary medical decisions.

Like all quantitative approaches this study had the ability to miss interesting facets. Thus, the authors suggest to evaluate and validate appropriate items for additional factors by means of qualitative interviews with pet owners. Re-evaluation of the items of the *Need for Alternatives* factor appears appropriate because its factor loadings and reliability were slightly smaller than in the other latent constructs and their indicator variables. The adaptation of validated tools to measure patient satisfaction from human medicine seems to provide a reasonable approach (84, 85).

#### Implications for Further Research and Practical Application

The study results suggest that the field of social sciences provides highly interesting opportunities to better understand the complexity of challenges and conflicts in daily veterinary practice. Results lead to the conclusion that there seem to be measurable interrelationships between underlying latent factors of RCC (empathic communication and partnership-building) during veterinary appointments. They not only seem to be closely linked with each other, but also show to have the potential to decrease the pet owners' feelings of not having all his or her questions answered. This corresponds to the knowledge available in previous human medicine and veterinarian studies. Within this study, items that were derived from human medical measurement tools to measure SDM (39) showed high factor loadings on the *Partnership-Building* factor (V38, ß = 0.90; V17: ß = 0.85; V13: ß = 0.82; V24: ß = 0.81; V16: ß = 0.79; V19: ß = 0.79; V12: ß = 0.78) and an excellent reliability. Therefore, an adaptation of human medicine measurement tools to describe SDM in veterinary medicine seems to be a promising approach to accelerate scientific progress in this field. Because individual items showed to be ambiguous in the context of veterinary medicine during the pretests (e.g., “to make a decision” got spontaneously associated with “euthanasia” by nearly all pretesting pet owners), item adaptation should not be done without cognitive pretesting to ensure validity.

Further, the findings suggest that empathic communication and partnership building could result in a decreased need of the pet owner to consult alternative medical services such as physiotherapists or homeopathic practitioners. An unfulfilled need for further information on pet owners' side can therefore be regarded as a potentially meaningful factor of the increasing reorientation toward complementary pet health providers in Germany (82).

A remaining question is whether a higher amount of information provided decreases the pet owners' needs for further information just directly or if there might be other latent factors that need to be considered. Assuming the latter, it may be hypothesized that the “act of information giving” results in a feeling of being cared for and therefore positively influences underlying psychological effects of trust building and decreasing fears, and uncertainties. The positive influence of providing information therefore might be direct as well as indirect.

One point that has to be turned out clearly is that RCC reaches for a balance of power between veterinarians and pet owners during the decision-making process. Imbalances in both ways—either a paternalistic approach with power on the veterinarians' side, or a “customer-like” attitude in pet owners reducing the veterinarian profession to that of a provider—increase the risks of dissatisfaction and inefficient animal health care. Therefore, such currents should be faced with appropriate critical caution. To denote patients or pet owners as “clients” or even “customers” and doctors or veterinarians as “providers” seems to be an increasing habit in human as well as in veterinary medicine. Regarding to Hartzband and Groopman (86) this habit is disruptive for the sensible interpersonal relationship during a medical consultation. It influences self-perception and behavior and results in dependencies and false expectations. Pet owners'—as well as patients'—expectations on the “product” or “service” of medical treatment and care prevent a realistic assessment of medical services and could be a source of the growing number of complaints. Cultivating this parlance and the resulting behavior patterns may not only have negative impact on a specific veterinarian-patient-relationship. It could also undermine the profession's self-perception and negatively influence the public reputation of the veterinarian profession at the societal macro-level (86, 87).

While the business aspect cannot to be completely ignored in veterinary practices, the modern principles of user-centered approaches in a way reflect the characteristics of RCC and may offer innovative solutions. These principles base on building up empathy with the users resp. pet-owners. Empathy helps to understand the individuals need in depth and therefore may lead to more successful businesses and rewarding working conditions (88). Such strategies should be combined with a confident self-perception of veterinarians that regard themselves as highly qualified partners in veterinary medical care. With regard to our study results, user- resp. pet owner-centered business approaches should be further investigated for their potential added value in veterinary medicine. For the moment the communication and decision making techniques given in the Calgary Cambridge Guide and SDM-schemes seem to offer user friendly guidelines to positively influence consultations that easily may be implemented to daily veterinary practice.

Although the study results allow a promising outlook on how interpersonal skills may positively shape the future veterinarian-pet owner-relationship, a huge amount of unanswered questions still remains in the field of veterinarian social science. Living and working in the “century of the patient” (89) requires to find answers—building strong partnerships in veterinary decision processes may be one of them. Being nice may not suffice—but it appears to be a good starting point.

## Ethics Statement

Within the study no personal nor sensible data were collected. Participation was voluntarily and anonymous. Before starting the questionnaire, participants perceived detailed information about the aims of the study, which data will be collected and how the data will be evaluated. Consent needed to be given actively by each participant. For no personal rights nor any German and European data protection laws could be violated, we refrained from receiving approval of an ethic committee.

## Author Contributions

AK conceived and designed the study, developed the theoretical framework, and implemented it into the preliminary model and questionnaire. Statistical preliminary considerations as well as coding the SAS code was done in close cooperation with RM. AK drafted and revised the paper. RM supervised and supported the project in each point of the development, conduction, statistical evaluation, and during the paper writing process.

### Conflict of Interest Statement

AK was temporarily employed in a Start-up business with interest in Digital Animal Health Care (vetevo GmbH) that potentially could have been interested in the study results. The employment relationship started almost 1 year after the start of the research project and ended before publications were done. The company was not involved in any steps of study design, data collection or evaluation, and no data or findings were provided to the company. Potential conflicts were prevented by obligation toward the privacy statements as well as the policies of good scientific work of the Institute for Veterinary Epidemiology and Biostatistics. The remaining author declares that the research was conducted in the absence of any commercial or financial relationships that could be construed as a potential conflict of interest.
